# Health-endangering everyday settings and practices in a rural segregated Roma settlement in Slovakia: A descriptive summary from an exploratory longitudinal case study

**DOI:** 10.1186/s12889-017-4029-x

**Published:** 2017-01-28

**Authors:** Andrej Belak, Andrea Madarasova Geckova, Jitse P. van Dijk, Sijmen A. Reijneveld

**Affiliations:** 1 Kosice Institute for Society and Health, Faculty of Medicine, P. J. Safarik University, Kosice, Slovakia; 2 Department of Health Psychology, Faculty of Medicine, P. J. Safarik University, Trieda SNP 1, 040 11 Kosice, Slovakia; 3 Olomouc University Society and Health Institute, Palacky University Olomouc, Olomouc, Czech Republic; 4 Department of Community and Occupational Medicine, University Medical Centre Groningen, University of Groningen, Groningen, The Netherlands; 5 Department of General Anthropology, Faculty of Humanities, Charles University, Prague, Czech Republic

**Keywords:** Slovakia, Roma health, Health inequality, Social determinants of health, Public health, Ethnography

## Abstract

**Background:**

Research into social root-causes of poor health within segregated Roma communities in Central and Eastern Europe, i.e. research into how, why and by whom high health-endangering settings and exposures are maintained here, is lacking. The aim of this study was to assess the local setup of health-endangering everyday settings and practices over the long-term in one such community. It is the initial part of a larger longitudinal study qualitatively exploring the social root-causes of poor Roma health status through the case of a particular settlement in Slovakia.

**Methods:**

The study, spanning 10 years, comprised four methodologically distinct phases combining ethnography and applied medical-anthropological surveying. The acquired data consisted of field notes on participant observations and records of elicitations focusing on both the setup and the social root-causes of local everyday health-endangering settings and practices. To create the here-presented descriptive summary of the local setup, we performed a qualitative content analysis based on the latest World Health Organization classification of health exposures.

**Results:**

Across all the examined dimensions – material circumstances, psychosocial factors, health-related behaviours, social cohesion and healthcare utilization – all the settlements’ residents faced a wide range of health-endangering settings and practices. How the residents engaged in some of these exposures and how these exposures affected residents’ health varied according to local social stratifications. Most of the patterns described prevailed over the 10-year period. Some local health-endangering settings and practices were praised by most inhabitants using racialized ethnic terms constructed in contrast or in direct opposition to alleged non-Roma norms and ways.

**Conclusions:**

Our summary provides a comprehensive and conveniently structured basis for grounded thinking about the intermediary social determinants of health within segregated Roma communities in Slovakia and beyond. It offers novel clues regarding how certain determinants might vary therein; how they might be contributing to health-deterioration; and how they might be causally inter-linked here. It also suggests racialized ethnically framed social counter-norms might be involved in the maintenance of analogous exposure setups.

**Electronic supplementary material:**

The online version of this article (doi:10.1186/s12889-017-4029-x) contains supplementary material, which is available to authorized users.

## Background

The Roma present the largest, internally most variable and traditionally most marginalized ethnically defined minority population in Europe. According to conventional social-scientific criteria, summing up of all the involved subgroups under one ethnically framed label “Roma” is problematic [1, 2]. Despite their shared common ancient ancestry on the Indian subcontinent [3], Roma subgroups show much greater variability in most tangible aspects, including e.g. their ethnonyms and mother tongues [4, 5], social organizations, customs, mutual relations [1, 6] and genes [3, 7], than subgroups of other ethnically defined European groups (such as the Dutch or the Slovaks). However, in their home countries the varied Roma subgroups constitute national Roma minorities, which alike occupy the lowest societal positions (e.g. attaining the lowest rates of employment, levels of education and income, the worst health status) [8–10] and which have historically faced and continue to face similar ethnically framed external pressures (e.g. discrimination, racism or outright antiziganism) [11–13]. Many social scientists claim that commonalities among the different Roma subgroups also involve similar ethnically framed ideologies and practices on their own part, albeit for the most part ones closely related to the external pressures mentioned [14–16].

As elsewhere in Central and Eastern Europe (CEE) [17, 18], compared to the general population, the health status of Roma in Slovakia appears to be consistently poorer, too. The worst health outcomes are shown for physically segregated communities, home to approximately 40% of 450,000 Slovak Roma. For these places, numerous surveys claim worse self-rated health e.g. [19, 20], demographic projections report higher mortality rates and a shorter life-span e.g. [21], and clinical studies show a significantly greater communicable and non-communicable disease burden across the life-course e.g. [22–27].

These segregated communities’ poor health outcomes seem to result from adverse circumstances therein. Higher smoking rates, less physical activity, riskier dietary habits and greater perceived healthcare access barriers have all been found in rigorous comparative studies e.g. [28–31]. Other research indicates poor community and personal hygienic standards, a missing or dysfunctional basic infrastructure, increased environmental hazards, overcrowding and even food shortages e.g. [26, 32, 33]. The only exceptions are findings debunking myths about higher alcohol consumption rates [28, 34], greater promiscuity [35], more adverse peer pressure [36] and dysfunctional social support [37, 38].

Research into the social root causes behind such and similar high health-exposures CEE Roma face is lacking. According to contemporary epidemiological theory [39–41], all steep ethnic health-inequalities result from complex and, at least in part, historically unique social processes. Such inequalities form when varied actors contribute through their acts and everyday practices to systematically different health-endangering exposures in ethnically defined populations. The involved kinds of actors typically range from global, national and local authorities to members of the populations concerned, but their actual compositions and contributions are historically contingent and transient. In order to understand what could be done to tackle a specific ethnic health-inequality, one thus also needs to study empirically how and why particular actors co-maintain specific related health-endangering exposures over the long-term – the social root causes of the inequalities. Such research is lacking in regard to CEE Roma [17, 18, 42, 43].

For research into the social root causes behind any particular health-inequality, qualitative case-studies focusing on the worse-off population’s health-endangering everyday settings and practices represent a good starting point. All disproportionate damage caused to the very bodies making up any worse-off populations happens exactly via the population members’ everyday settings and practices [44, 45]. Focus on this intersection in turn enables the tracking of all involved actors, whether local or distant [46, 47]. It also enables identification of the nature of these actors’ negative local influences, including their complex local mutual interplays [48, 49]. Especially where health-related everyday settings and practices are not well known – such as for CEE physically segregated Roma – examination of particular, carefully-selected cases using intensive qualitative methods is a relatively cheap and logistically modest explorative strategy. Specific causal pathways worth further examination in the specific context can thus be conveniently identified (or discovered) [47, 48, 50].

Here we present a study aimed at assessing the local setup of health-endangering everyday settings and practices over the long-term in a segregated rural Roma settlement in Slovakia. It is the initial part of a larger longitudinal study qualitatively exploring the social root-causes of poor CEE Roma health status through a particular case.

## Methods

### Design

**Fig. 1:**
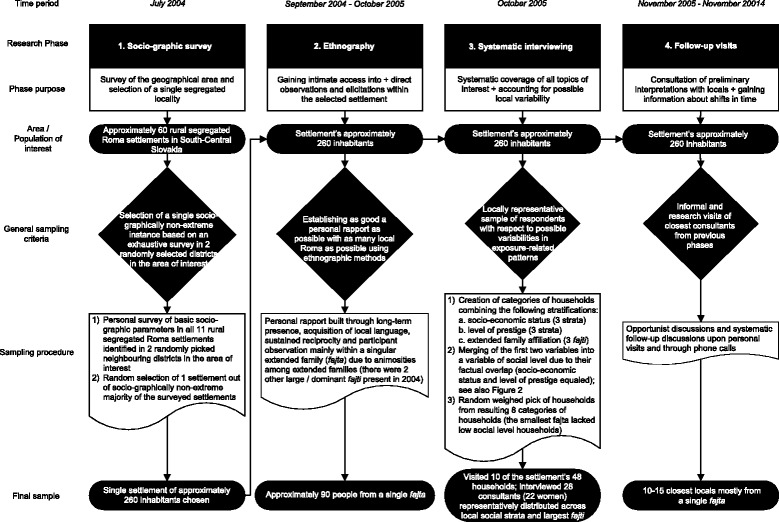
Sampling procedures. Scheme specifying the four methodologically distinct phases of the study

The study comprised four methodologically distinct phases (see Fig. 1). It combined ethnography (phases 2 and 4) [51, 52] with methods used in applied medical-anthropological surveying (phases 1 and 3) [53, 54]. First, a socio-graphic survey of several localities was carried out in order to select a single segregated place. Next, ethnographic methods were used in the selected place to gain close personal access to and primary data regarding the setup and possible social root-causes of the local everyday health-endangering settings and practices. Consequently, systematic interviewing was undertaken to increase local representativeness and the systematic breadth of the collected material. During the last phase, local people’s reflections of preliminary interpretations and additional material regarding long-term shifts in local health-endangering settings and practices were constructed through follow-up communication.

The fieldwork was carried out by the corresponding author. Acquired data consisted of field notes on direct participant observations and records of personal elicitations focusing on both the setup and possible social root-causes of local everyday health-endangering settings and practices. To gain data specifically and exhaustively regarding all aspects considered to be health-endangering according to contemporary biomedical theory, throughout all phases of the study an encyclopaedic practitioner’s handbook covering both clinical and public-health knowledge was being used to guide observations and elicitations [55].

### Settings and samples

The south-central region of Slovakia was picked because of its historically high proportion of segregated Roma residents [56]. The single settlement used in this study, selected based on the socio-graphic survey, had a growing population of approximately 260 people (230 in 2004, 300 in 2014) – all self-declared Roma and speaking Romani as their mother tongue – compared to a declining population of approximately 530 non-Roma living in the rest of the village (580 in 2004, 470 in 2014). In 2004, approximately half of the settlement’s inhabitants were children under 15 years old, and only 5 people were older than 60. For a concise overview of the recent history and variability of segregated Roma Settlements in Slovakia, see Scheffel [57] and Musinka et al. [56].

**Fig. 2:**
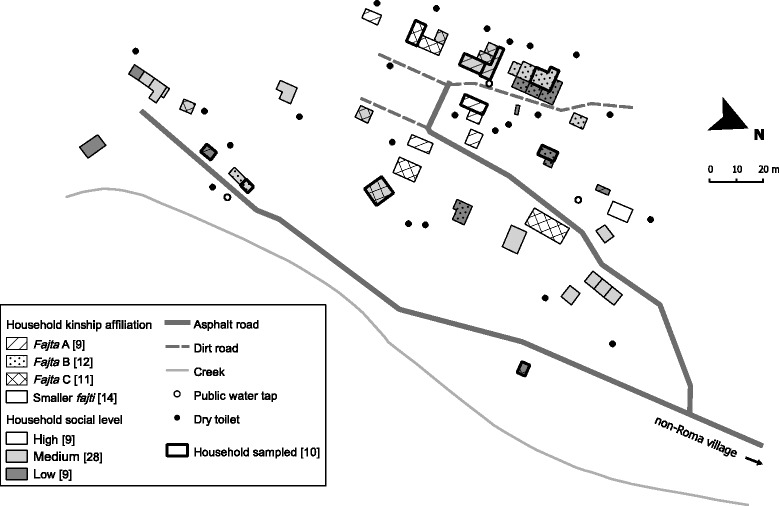
Map of the Roma settlement. Schematic map of the settlement also depicting stratifications used in the study; particular *fajti* refer to local Roma kinship formations (see main text for further details)

The sampling is detailed in Fig. 1 and Fig. 2. In the first ethnographic phase, most data obtained in the settlement was related to approximately 90 people belonging to one of the 3 then largest local *fajti*, i.e. specific transient kinship formations roughly overlapping with unilateral extended families [58]. The systematic interviewing visited a sample of 10 households out of the settlement’s total 48. The sample was representative according to the households’ local socioeconomic position (SEP), level of prestige, and affiliations to *fajti*. In the selected households, 28 informants were elicited, with 22 of them being adult women. Several other people participated in shorter sequences of the interviewing. Locally, men were considered less competent regarding health-related issues both by themselves and by women, and most of them also showed less interest in discussing health spontaneously. None of the people approached refused to participate in the interviewing. The closing follow-up observations and elicitations were limited to approximately 15 locals personally closest to the corresponding author.

### Procedure

*The socio-graphic field survey phase* consisted of personal inspection of all existing 11 rural segregated localities in the surveyed area. Local demographics, infrastructure, history, socioeconomic differences and inter-ethnic relations were assessed using brief questionnaires (see Appendix). The particular settlement was selected randomly from the majority of examined localities that exhibited non-extreme characteristics in all surveyed respects: middle-sized enclaves older than five decades, physically and socially segregated, with substandard public infrastructure and apparent internal socioeconomic gradients.

*The initial ethnographic phase* consisted of establishing personal rapport with the local Roma, acquisition of proficiency in their primary language (a South-Central dialect of Romani) and in participant observation of their settings and practices. In this phase, the researcher’s focus on the bio-medical aspects remained opportunistic and unsystematic.

The *systematic interviewing* was carried out using a bilingual template of implicit topics covered by several hundreds of questions in local Romani dialect, with particular questions focusing on elicitation of the locals’ perspectives on the local setup and the social root-causes of particular local health-endangering settings and practices. The direction and sequence of questions for each particular topic were identical and analogous to the logic of elicitation proposed by Arthur Kleinman and widely used in clinically applied medical anthropology to construct ‘illness explanatory models’ [59, 60]. Particular topics were adopted from the practitioner’s guide [55]. Wording was prepared with a close local informant. Interviewing differed considerably between households in detail and depth within a length range from several hours to several days per interview. The corresponding author’s rather specific position of a friendly outsider and a supposed health expert allowed him to interview adult women intimately despite being an unrelated adult male – i.e. exceptional, according to strict local gender norms. Answers to questions were recorded in writing with a focus on capturing parts considered directly relevant to the particular questions and the specific Romani expressions used. Stratifications of households used in the sampling of households (see Fig. 1 and Fig. 2 for details) represented the consensus of several local informants regarding the particular households’ SEP (*barvaľipe* = affluence; associated with possession of amenities), level of prestige (level of ascribed *gizda* = snobbery), and affiliations to extended families (*fajti*).

*The follow-up ethnographic communication* was carried out through visits of the settlement regularly until late 2010, ranging from several days to several months in length. Until late 2014, regular follow-up elicitations continued with the locals over the phone and in person outside the settlement. In addition to written field-notes on observations and informal elicitations, semi-structured in-depth follow-up interviews were organized and recorded by the corresponding author on several occasions.

### Coding, analysis and reporting

To summarise the study findings on the local setup of health-related settings and practices, we coded and analysed selected study data as described in detail below. To ensure comprehensiveness and convenient intelligibility of the summary, especially for public health practitioners, we based the analysis on the latest World Health Organization (WHO) classification of known health-exposures, defined under the notion of ‘intermediate social determinants of health’. The classification comes from a widely used theoretical framework, the WHO Framework on action for social determinants of health [45], compatible with our theoretical premises regarding the social root-causes of health-inequalities cited in the Background.

We coded and merged all types of data from all sources and phases of the research as follows. We first coded any sequences of field notes considered relevant regarding the local setup of health-endangering settings and practices as such. Field notes from the initial ethnographic phase and from the follow-up ethnographic communication were coded manually, while transcripts of audio recordings from the late interviews were coded using the MAXQDA® software. To these sequences of text, as well as to those parts of the systematic interviews explicitly covering analogous themes, we then ascribed further hierarchical codes to distinguish data sequences relevant for particular domains of exposures and for their core elements, as defined in the guiding WHO source [45]. In parallel to all these sequences of text, we also assigned codes denoting their relevance regarding the following variables: SEP (codes for ‘rich’, ‘common’ and ‘poor’ households), level of prestige (codes for ‘snobby’, ‘normal’ and ‘squalid’ households), affiliations to dominant *fajti* (codes for families ‘A’, ‘B’, ‘C’ and ‘smaller *fajti*’), and time period (codes for ‘first three years’, ‘mid-period’ and ‘last three years’). The same sub-coding was also applied to the selected relevant parts of the systematic interviews. As the levels of SEP and levels of prestige factually equalled – e.g. the households ascribed highest socioeconomic position were also ascribed the highest level of prestige – upon coding we eventually merged these two variables into one entitled ‘social level’. This variable had three levels – high, medium and low – each indicating the levels of both SEP and prestige ascribed to particular households (see also Figs. 1 and 2).

We then performed qualitative content analysis, combining all coded data from the field notes and the relevant data from the systematic interviews, i.e. on health-endangering settings and practices. As a method for content analysis we used recurrent abstraction [61]. This means that we repeatedly read and in steps summarized all text sequences on the endangering settings and practices that the locals faced and on how they engaged with them, regardless of their original source. Upon summarizing, we focused mainly on capturing the variability and dominant trends in local health-endangering settings and practices. For each intermediary determinant, we first created descriptive summaries regarding its particular core elements for particular social strata. We then cross-compared these summaries for estimations of major differences according to social level. To assess variability with respect to distinct age, gender and family-affiliation groups as well as with respect to time periods, notes were taken during the process of re-reading and then summarized for each domain of social determinants separately.

Based on the above-described analysis, we report on the local setup of health-endangering settings and practices across the following intermediary social determinants of health, as defined in the WHO source [45]: material circumstances, psychosocial factors, health-related behaviours, social cohesion and health-system interactions. For each, we present dominant local trends regarding particular core elements. To these observations, we add notes on related variability according to social level, age, gender and research periods (no variations were found across *fajti*). We used bold text to point to particular core elements of the discussed intermediary determinants. To support the thus constructed findings with original data, we include illustrative quotes by local consultants. The quotes were selected based on two criteria: 1) they compactly illustrate our findings regarding particular exposures in the locals’ own wording, and/or 2) they compactly illustrate on what kind of utterances we base our observations, suggestive of the locals’ racialized ethnically framed reasoning (See also Additional file 1: Fieldwork visual reference for illustrative photographs).

## Results

### Material circumstances

The majority of *houses* in the settlement were built illegally and maintained in a provisory way using unsuitable materials such as industrial landfills waste. Most houses sheltered several separate households in improvised extensions of the original buildings. Typical *internal housing conditions* included over-heated and damp air (locals’ comfort zone was in the upper 20s°C and most houses were not ventilated regularly), cold walls (no insulation) and overcrowding (rooms sleeping up to five people were common).

Most households lacked basic *household infrastructure*. Only two households had indoor running water. For both heating and cooking, raw wood, illegally harvested from surrounding forests, was being burnt in second-hand iron stoves. Most households were connected to electricity, but many only through illegal extensions via other households. Several households used reclaimed car batteries instead. Only one nuclear family possessed a bathroom. Everybody used self-built outdoor dry toilets, with most children up to six years old defecating in public spaces. Basic *amenities*, such as refrigerators, washing machines, audio-visual entertainment equipment and cars, were popular and common in the settlement but usually limited to second-hand items which did not work and were not used as intended by the manufacturers. ‘Strong’ hi-fi equipment and cars in particular were praised and preferred as ‘Gypsy’ features.

Apart from the electrical network, *community infrastructure* consisted of one asphalt road connecting the settlement to the nearby village, several dirt roads and three outdoor sources of cold potable water (see Fig. 2). For liquid-waste disposal, households with running water used improvised drainages out to public spaces; others used the surroundings of their houses. Solid waste was being burnt in public places, disposed of at improvised landfills on the outskirt of the settlement or in open industrial containers provided and occasionally emptied by the municipality.

Related *direct health-risks* included: regular health and safety incidents within households (e.g. roof implosions, leakages, fires, window breakages); unhealthy household climates; frequent electricity outages and occasional injuries from improper handling of equipment; lack of personal-hygiene means, the presence of parasites (e.g. lice, fleas) and frequent intestinal infections; contamination of public space by urine, faeces and smoke, the presence of rodents; constant ergonomic strain. Related *social and economic tolls* included: stigmatization outside the settlement (due to e.g. smell, parasites, dirty clothes, outworn equipment) and frequent relatively high-cost breakages (due to e.g. high-input and short working life of the outworn equipment).

The highest-ranked households occupied old, legally built former peasant houses, possessed amenities in better condition (e.g. registered cars, chainsaws, DVD-players), and paid more attention to tidiness outside their households (e.g. possession of a waste bin, children using dry toilets).

Over the 10-year research span, some material aspects improved, especially for those holding higher social positions. Several highest-ranked households insulated their houses; two bought new washing machines; most families originally in the possession of a car (some medium and all high social level households) kept renewing their second-hand fleets; several high and medium social level families purchased reclaimed computers, and even the poorest families were able to start using newer models of second-hand mobile phones. The lowest-ranked households experienced little or no improvements, with some of them experiencing further decline (e.g. moving from a deteriorated self-built wooden shelter to a smaller reclaimed wagon).

For illustrative quotes related to the above section of Results, see Table 1.

**Table 1:** Material circumstances quotes. Quotes illustrating our findings regarding local health-endangering settings and practices (primarily material circumstances related) and the local consultants’ related ethnically framed reasoning

Quotes	Exposure elements
‘*I wish they* [own children] *would have more money than us… Why? So they’re not down like us, so they don’t have to steal wood, recycle metal.*’ A., woman, 34, low social level [Sep 2005]	Household infrastructure; SEP
‘*Do you know why Roma have always preferred the Žigulis over the Škodas* [car brands]? *Because of their acceleration! A Gypsy needs his engine to roar, you know what I mean?*’ Z., man, 37, medium social level [Jul 2005]	Amenities
‘*Of course it could be from the water [frequent diarrhoeas]. You’ve seen how we pulled water towards M’s house. It’s the same as with electricity and everything here. You want a new connection? You make it yourself* [laughing]. [AB: But don’t at least the local public water taps get checked for quality? I asked around and they do this regularly in the village.] *C’mon, nobody like that* [public health authorities] *would ever come up here.*’ S., man, 32, medium social level [Sep 2005]	Household infrastructure; Community infrastructure; discrimination
‘*What’s there not to like about it?* [about rubbish in public spaces] *This is normal here. We are Gypsies* [sic] *so we live like Gypsies.* […] *You don’t have to eat from the ground!*’ M., woman, 36, high social level [Jun 2010]	Community infrastructure
‘*Ok, they* [the municipality] *built this road here back then. When you are in need of Gypsy votes* [for the mayor elections], *everything is possible! But imagine you live back there* [in the part of the settlement not connected to the asphalt road] *like P.* [low social level cousin]. *No matter what you do, once it rains, you’re all mud. And now go and visit the paediatrician.*’ K., man, 48, medium social level [Jul 2010]	Community infrastructure; social tolls; healthcare use

In the adjacent column we list the exposure elements discussed

### Psychosocial factors

In terms of *longer periods of stress*, the locals appeared to suffer the most from their nuclear family members’ detachment due to hospitalization, work trips or incarceration, and an effort was made to prevent such scenarios. Another long-term stressor was the terminal stages of terminal diseases in related elderly. The locals perceived the following in particular as the most frequent *incidental stressors*: a total lack of funds (i.e. fluctuating periods of literally no cash and no subsistence opportunities for the coming several weeks), visits outside the settlement beyond the nearby village (approximately weekly trips to the local administrative centre) and incidents of physical violence (approximately monthly involvement in fights within the settlement, mostly due to jealousy or conflicts of interests amongst *fajti*, culminating during celebrations after welfare payments).

Based on assessments of local informants (including non-Roma villagers), the settlement’s Roma inhabitants had a radically lower *socioeconomic position (SEP)* compared with non-Roma living in the nearby village. Only one man in the settlement was long-term employed (as an industrial-construction worker), while most others worked only seasonally (and mostly illegally while formally unemployed) as occasional labourers, typically in construction or agriculture. A few high-ranked Roma women worked as unqualified helpers either in agriculture or (in latter periods) in services. The regular income of most Roma families depended on social-welfare payments, recycling (mostly of scrapped metal), and in the case of the lowest-ranked households also on gathering, hunting (e. g. mushrooms, fish), and petty thefts (poultry, cigarettes, cash). Several older higher-positioned Roma people held apprenticeship certificates acquired during the Communist era, and several younger people were studying to earn one, too. Most people, however, dropped-out of all formal-education trajectories early, and the majority had only finished compulsory elementary-school attendance.

*Perception of own low SEPs* (both within the settlement and in comparison, to local non-Roma) did not appear to cause the locals any stress. The low SEPs nevertheless remained causally linked to the frequency of a total lack of funds, which was considered an incidental stressor. Common *experiences of racism and discrimination* (e.g. preferential treatment of non-Roma in GP waiting rooms or withdrawals of employment opportunities upon arrival in person) were considered unjust but ‘normal’ and not stressful unless overtly offensive.

We identified the following local strategies to prevent and cope with stress. These were solidarity within *fajti* in cases of food shortages, serious health issues and violent incidents; solidarity beyond *fajti* where children were at serious risk (e.g. rides to the hospital) or in conflicts with non-Roma (e.g. side-taking in fights); travelling only in groups pretending ostentatious confidence (e.g. loudness, strict speech-tones); avoidance of persons and institutional venues with a racist track-record; and drawing self-assurance from adherence to social norms framed in ethnic terms (‘Gypsy’ / ‘Romani’) constructed and praised mostly in contrast or in direct opposition to alleged non-Roma norms. The above strategies were not being interpreted by the locals themselves as such.

The local ethnically framed social counter-norms included e.g. specific work ethic (stressing the importance of the Roma ability to improvise in contrast to the non-Roma ability to bear drudgery), a specific kinship ethic (stressing e.g. different concepts of discretion and openness between generations), aesthetic style (e.g. relatively expressive ‘Gypsy’ clothing patterns and festive verbal communication), etc. The appropriateness of the locals’ adherence to the particular norms was often argued by them using racialized arguments quoting specific related ‘natural’ Roma capacities embodied via their ‘blood’, ‘brains’, ‘bodies’, ‘genes’, etc. The same or analogous reasoning and argumentation was commonly used in local discussions (i.e. also beyond the discussions with the researcher) of most local practices (for examples from other domains see other sections of Results).

Across local social strata, the only variability in any of the above concerned that higher-ranked people experienced more stress regarding financial difficulties projected over longer periods (e.g. related to pension plans). Moreover, some practices necessarily associated with the maintenance of a higher SEP, such as compliance with rules in education, long-term employment, etc., were generally considered and ridiculed locally as being ‘too non-Roma-like’ (*gadjikano*). This posed an extra psychosocial dilemma for locals of high social level. In turn, lower-ranked people were generally considered and respected as more ‘true Roma’ (*prave Roma*). Over the last five years of the study period, a general lack of means of subsistence seemed to become more severe and continuous (constantly decreasing seasonal, unqualified and unregistered employment-opportunities; decreasing welfare payments; increasing formal requirements for employment; increased indebtedness by commercial lenders). This shift was also quoted as the sole reason for alleged local dramatic increase in the use of prescription tranquilizers and antidepressants in the settlement.

For illustrative quotes related to the above section of Results, see Table 2.

**Table 2:** Psychosocial factors quotes. Quotes illustrating our findings regarding local health-endangering settings and practices (primarily psychosocial factors related) and the local consultants’ related ethnically framed reasoning

Quotes	Exposure elements
‘*I am never-ever going to sit there on behalf of Fat Face again* [crying, talking about a humiliating experience from a municipal committee meeting chaired by the village mayor]. *When I start talking there, you know, I cannot put my words together as well as the others. I know what I would like to say, but I just don’t speak non-Romani as well as they do. And then the looks – look at the stupid Gypsy speaking…*’ M., woman, 31, high social level [June 2005]	Stressors; stress-coping and prevention strategies
*‘This is the hardest thing both for him* [referring to husband and father of four away at a rehab stay] *and us* [his nuclear family and siblings]. *We don’t see him every day, we can only visit him now and then thanks to M* [a better off sister]. *He’s among the non-Roma all by himself all the time. The terrible silence, the watery food. Oh, God, I really think it would be far better for all of us if he just stayed at home and drank himself to death here!’* K., woman 43, medium social level [Jul 2005]	Stressors; stress coping and prevention strategies; bonding social capital
‘[To us, enough money is] *when you simply don’t need any more of it… When I imagine I’d have to work all the time like some non-Roma… they just work and work like dummies, then they fear for their money… I only need what I already have… I would only like to have the same a bit more easily.*’ D., woman, 31, medium social level [Aug 2005]	SEP perception
‘*There’s so many disgusting things you non-Roma do! I cannot imagine my daughter seeing me without pants.* […] *Or look at how you don’t fear anything, the dead, the pain* […] *your hearts are made of stone.*’ M2, woman, 29, high social level [Jul 2010]	Stress coping and prevention strategies; bonding social capital
‘*I tell you why* [many Roma nowadays visit psychiatrists for subscription medications]. *Because these are the hardest times we’ve ever been through. It has never been this bad before. Everybody can feel nothing good is coming our way anymore. Women are afraid for their kids’ future.* [Seasonal] *work for men is gone. We will now even be working for free* [referring to a new unemployment law]!’ S., man, 41, medium social level [Aug 2014]	SEP; SEP perception; healthcare use

In the adjacent column we list the exposure elements discussed

### Health-related behaviours

The majority of adult and teenage men and the greater half of adult women in the settlement were daily *smokers*. Teenage women and younger people were being discouraged from smoking by others and lacked finances to purchase cigarettes regularly. People preferred to smoke high-priced labels of cigarettes but usually could afford only hand-made tobacco cigarettes without filters. Adult women and teenage women smoked more privately and less often; in the lowest strata children of all ages smoked occasionally.

Similar age and gender patterns were present with respect to *alcohol consumption*. Above the lowest social strata, where binge drinking was somewhat more frequent (several times per month), daily drinking was only moderate for most of the month (a pint of beer now and then) with the exception of two alcohol-dependent persons. In most households, binge-drinking took place solely following monthly welfare-payments and at anniversary celebrations. Even here, however, drinking until loss of basic social skills (more common amongst non-Roma men in the nearby village) was being discouraged as inappropriate. Such celebrations included loud reproduced ‘Gypsy music’ and intense dancing. During the research period there were no other cases of *drug abuse* observed.

*Promiscuity* was being strongly discouraged by everybody with respect to adults and teenage girls. Promiscuity of young men was being encouraged and praised, however, especially in a direction outside the settlement, including engagements with non-Roma women. Beyond such rhetoric, most pre-marital sexual relationships appeared as local and opportunistic, with women and men sharing their experiences only privately and with both sexes allegedly having only occasional pre-marital intercourse. Marital adultery was supposed to be common (several incidents per life per person) but was heavily sanctioned (e.g. public beatings or temporary retreat/expulsion of spouse to his/her parents’ house) and never admitted publicly. Higher-ranked families put more effort into preservation and display of a non-promiscuous history of their teenage girls.

As regards *consanguinity*, people typically tried to form couples across geographical distance, yet preferred partners from already related *fajti*. Several first-cousin marriages were thus tolerated, silently or stressing the families’ distant residency. High and medium social level adult women often opted for *intrauterine devices* after conceiving several children. *Condoms* were not in use in the settlement.

The local *diet* consisted mostly of large portions of cereal-flour based meals (bread, home-made fresh ‘Gypsy pasta’ and regular pasta) combined with potatoes, rice, cheap meat (smoked sausages, poultry and fatty pork), and cooked vegetables. Most people did not consume any fresh vegetables, fruits or nuts. On more prominent occasions (Sundays, celebrations), unhealthy foods such as roasted fatty pork-meat was served preferably. Whenever possible financially, i.e. mostly according to social level, children were constantly offered sweets and people were drinking high-sugar soft-drinks.

With the exception of winter months, when watching TV for several hours per day was common, most children and adults within the settlement engaged in strenuous *physical activity* throughout most of their days. Children were playing outdoors, men were busy with taking care of wood, repairs of all kinds (houses, cars, other amenities), and women with repetitive cleaning of households, washing of clothes, cooking and carrying around of children. In addition, most people frequently organized gathering and hunting hikes in small collectives for both additional subsistence and entertainment (collecting scrapped metal, picking mushrooms, harvesting corn or potatoes, catching crawfish). Given the lack of standard amenities for most activities in most households, all of the described physical activity was relatively *un-ergonomic* and often dangerous. With increasing social status, people could afford and readily invested more into amenities that made the respective activities more comfortable (e.g. purchasing second-hand chainsaws).

For illustrative quotes related to the above section of Results, see Table 3.

**Table 3:** Health-related behaviours quotes. Quotes illustrating our findings regarding local health-endangering settings and practices (primarily health-related behaviours related) and the local consultants’ related ethnically framed reasoning

Quotes	Exposure elements
‘*Of course I believe it's true* [that smoking causes cancer]. *So what? Nobody will ever make me stop anyway. A Gypsy* [sic] *will always only want to live like a Gypsy… this is his life, to have a little smoke, to have a little fun, to drink a little.*’ S., man, 31, medium social level [Jun 2005]	Smoking; alcohol consumption
‘*This is what being a Gypsy means, if it rains, you get wet, you work away, you won't quit and change* [your clothes]. *When you're finished, only then you can change… This is what we are used to… Sure, you catch a cold - but you get healthy again! When you're in the middle of something, just do it, you wash, you change, you eat only afterwards.*’ K., man, 27, medium social level [Sep 2005]	Ergonomic strain; risk perception
‘*Secretly, most women will have their fun before their wedding…* [Interviewer: ‘*Don’t they fear getting pregnant… since, you know, condoms are not used around here*’] …*A fear of what, new children being born? And what’s bad about that? When there’s a child, there will be a family… Usually, the two youngsters would really mean it once they don’t fear taking it this far. There are lots of families like that here and they live happily.*’ Z., woman, 25, medium social level [Sep 2005]	Promiscuity; contraception
‘*What is she* [talking about own daughter] *a whore to smoke this young?* […] *And tell me where would they get money for that* [talking about occasional smoking of young children in the settlement]*?*’ S., woman, 30, high social level [Sep 2005]	Smoking; SEP; social norms
‘*Gypsies love meat, especially pork meat, everybody knows that.* […] *And we hate thin food with no taste. The soups you eat, pure water!*’ M2, woman, 27, high social level [Sep 2008]	Diet

In the adjacent column we list the exposure elements discussed

### Social cohesion

In most respects, *bonding social capital* within the settlement was restricted to *fajti* comprising of several dozens of people. Despite a simultaneous public performance of distance according to the ascribed levels of prestige (hierarchies running within particular *fajti*), within these local kinship networks solidarity was absolute: from sharing of or donating supplies to nuclear families in need, through constant reciprocity of small favours, to side-taking in violent conflicts. Across local *fajti* boundaries, relationships were mostly competitive and rogue (constant mutual monitoring, blackwashing, public provocations, ostentatious ignorance, feuds, etc.) with the exception of life-threatening incidents involving children or elderly or in conflicts with non-Roma outside the settlement. Both of these bonding-capital networks provided an important *welfare safety-net* with respect to frequent health-related crises (e.g. preventing hunger, and assistance with severe injuries).

Framed in kinship (within *fajti*) and ethnic terms (towards the non-Roma), the above described *social norms* on one hand encouraged and enabled the building of strong local-bonding networks and on the other contributed to the isolation of particular *fajti* from each other and of the whole community from the non-Roma. The second restriction made it difficult for the locals to accumulate any *bridging* and *linking social capital* through engaging in reciprocity with outsiders. The lowest-ranked families possessed almost no relationships with non-Roma (except for friendships with socially excluded non-Roma), while even the highest-ranked individuals managed to maintain only several informal personal ties with local non-Roma from the village and only exceptionally in various local offices (e.g. long-term friendships or regular barters actively hidden from public by the non-Roma). The lack of bridging and linking capital meant the only resources the communities could rely on with respect to health were resources owned by the particular local *fajti*.

For illustrative quotes related to the above section of Results, see Table 4.

**Table 4:** Social cohesion quotes. Quotes illustrating our findings regarding local health-endangering settings and practices (primarily social cohesion related) and the local consultants’ related ethnically framed reasoning

Quotes	Exposure elements
‘*Yes, their house is cleaner* [referring to a low social level household from a different *fajta* in comparison to her own brother’s low social level household], *that’s true. But you can see it yourself, they’re just so stupid… they will always be the lowest ones. I’d definitely rather eat in my filthy brother’s house, at the Italian’s* [a nick-name]*, than at the Ds!’* D., woman, 31, medium social level [Jul 2005]	Bonding social capital; social norms
‘*Now if I had a kid, anyone from up there* [referring to more affluent people within the settlement] *would help me to get it to the hospital, you know that.* […] *Around here, I only know X* [reviewing non-Roma acquaintances]*; this guy living in his parents’ house on his own. Sometimes, I’d go visit him and we’d drink together. He was in jail, too, you know.*’ J., man, 37, low social level [Sep 2005]	Bonding social capital; social norms; linking and bridging social capital; welfare safety net
‘*You know, if you want to understand what Gypsy means, you should really talk rather with people like J.* [a low social level man living in a reclaimed wagon] *or P. and his wife* [a medium social level related family living in a self-made hut] – *they are proper Gypsies. You have to take care the* gadje [non-Roma] *way if you want a kitchen like this, if you want a washing-machine like this.*’ M., woman, 34, high social level [Aug 2008]	Bonding social capital; Linking social capital; SEP; amenities
‘*I don't understand how you non-Roma can let your little children be treated in schools the way they are being treated there. Strangers yelling at them… all the boring stuff… and you have to sit there and sit silently forever. Roma kids are unable to go through that… your kids are different.*’ M., woman, 36, high social level [Mar 2010]	Bonding social capital; linking social capital
‘[If their kids would grow-up at A.B.’s mother’s house], *it would still be the same for them, I think. Maybe some small things would change… for some time… but most of it would be the same. It’s in our blood! You know how they say’Gypsy blood’ – you cannot change that, no matter what.* […] *Our life is so much better, more colourful.*’ L., woman, 43, high social level [Sep 2014]	Bonding social capital; social norms; linking social capital

In the adjacent column we list the exposure elements discussed

### Healthcare utilization

Within the settlement, *access to healthcare services* differed along the social gradient. As elsewhere in Slovakia, services *availability* (the technical potential to deal with local health needs), *accessibility* (relative geographical distance) and *accommodation* (ability to meet the technical constraints of potential clients) met contemporary EU standards (i.e. at least since a major health reform introduced in 2006). Several general practitioners were available within a radius of some kilometres. There was a local hospital 15 km away with an emergency entry-point operating non-stop, and emergency-rescue teams were typically able to reach the settlement within 15 minutes of being called, as required by law. In households of lower social status, however, people experienced greater *affordability* difficulties. Most of the time, their members were incapable of paying for transportation and service-related complementary fees (e.g. purchase of prescription medications not fully covered by the national insurance plan). Only the highest-ranked people were able to visit the appropriate providers whenever they felt they should, though with periods when even they could not. Others would recourse to improvised home-healing or sometimes to inappropriate use of emergency services (e.g. emergency calls in cases of uncomfortable long-term stomach pain).

*Actual use* of healthcare services within the described affordability constraints was as follows. Regarding light transient diseases (*bežne nasvaľipna*), all local people used available services and took medication whenever possible exactly as recommended. Regarding serious chronic and terminal diseases (*phare nasvaľipna*), consistent following of clinical recommendations over longer periods was generally an exception. Analogous general non-compliance occurred also regarding preventative and recovery recommendations. Such *non-compliance* regarding serious chronic and terminal diseases contrasted with several local strong inclinations. Everybody in the settlement feared pain and death, often spontaneously describing such emotions as ‘naturally’ more intense in comparison with the non-Roma. All locals claimed interest in and made an effort to learn their diagnoses and related medical recommendations, especially regarding chronic and terminal diseases specifically. And all locals trusted local medical practitioners, especially in terms of the functionality of their medical know-how (often quoting also a supposed non-Roma ‘naturally’ superior capacity to ‘deal with complicated matters’). This contrast was considered understandable based on its congruency with local understanding of ‘proper (Roma) life’, framed in ethnic and often racialized terms. E.g. the locals would spontaneously quote a ‘natural Roma incapacity’ for long-term attentiveness to one’s health. Despite evocations of ‘nature’ and biology (‘Gypsy blood’, ‘Gypsy brains’, ‘Gypsy genes’) in such claims, however, in practice such ethnically framed norms applied mostly to the adult population and did not apply to children (at all) and elderly (as strictly). In the cases of the latter, healthcare was being utilized without any normative restrictions.

People of higher social status tended to opt for use of healthcare services for lighter symptoms – some of which the lower-ranked people did not regard as health issues – and to engage in short-term attempts to follow medical recommendations also regarding severe diseases. Among people of lower social status, *knowledge* and interest in knowledge of medical theory and recommendations was much rarer. Instead, people were using either explanations and therapeutic procedures improvised *de novo* within nuclear families (e.g. treatment of syphilis with petrol) or therapeutic procedures analogous to those used among local non-Roma (e.g. folk herbal-medicine).

For illustrative quotes related to the above section of Results, see Table 5.

**Table 5:** Healthcare utilization quotes. Quotes illustrating our findings regarding local health-endangering settings and practices (primarily healthcare utilization related) and the local consultants’ related ethnically framed reasoning

Quotes	Exposure elements
‘*Of course they* [healthcare staff in the nearby administrative centre] *treat non-Roma differently… but they don’t do any harm to us.*’ K., man, 27, medium social level [Sep 2005]	Discrimination
*‘What’s causing them, what’s causing them* [diseases in general]*… bacteria, right?* [A.B.: ‘And what’s that exactly?’]… *It’s these miniature* [sic] *animals… They live in the body, there are lots of them there; they eat and they destroy you with it… at least this is how I saw it* [in a TV documentary].’ D., woman, 31, medium social level [Sep 2005]	Medical knowledge
‘*You bet I’d see the doctor more often* [did she have more cash for public transport] *with my kids at least* […] *How would we take those* [prescription pills]*? We take them exactly as told* [by the healthcare staff] *sure, the way the hours are supposed to go* […] *Are you crazy? You cannot just add more to what you should be taking, that could hurt you… or it just doesn’t work then.*’ A., woman, 34, low social level [Sep 2005]	Healthcare affordability; SEP; healthcare use; compliance
‘*Most people here only make some effort* [taking medications for chronic diseases] *when they are in unbearable pain. A soon as the pain goes, they return to normal Gypsy life.* [AB: What do you mean?] *You know, we stop caring that much. You start smoking more, eating what you like and so on.*’ D2., woman, 34, lived in a nearby town, visiting a high social level sister [Jul 2005]	Compliance; social norms; bonding social capital
‘*We* [the Roma] *are like that. We cannot withhold pain. When we are in pain, we panic. But what can we do?* […] *I have tried to stay off chilli food for some time. I took the pills* [medications for oesophagitis]. *But, see, even I am not enough of a* gadji [non-Roma woman] *to stay that serious all the time. I will bear for some time. But then I just say to myself, what kind of a life is this? So you will vomit, so what?*’, M., woman, 36, high social level [Mar 2010]	Compliance; social norms; bonding social capital

In the adjacent column we list the exposure elements discussed

## Discussion

We conducted a longitudinal study aimed at assessing the local setup of health-endangering everyday settings and practices over the long-term in a segregated rural Roma settlement in Slovakia. It is the initial part of a larger longitudinal study qualitatively exploring the social root-causes of poor CEE Roma health status through a particular case.

We found that across all the examined dimensions – material circumstances, psychosocial factors, health-related behaviours, social cohesion and healthcare utilization – all the settlements’ residents faced a wide range of health-endangering settings and practices. How the residents engaged with and in some of these exposures and how these exposures affected whose residents’ health varied according to local social stratifications. Most of the patterns described did not change over the 10-year period. Our summary also conveys that some of the local health-endangering settings and practices were commonly praised by most inhabitants using racialized ethnic terms constructed in contrast or in direct opposition to alleged non-Roma norms and ways.

No other scientific studies of comparable depth have been published. However, all of our findings on material conditions, healthcare accessibility, health-related behaviours and living standards match what has been identified as typical for segregated settlements in Slovakia in the same period by non-governmental sociographic and sociological surveys e.g. [19, 20]. Regarding psychosocial factors, social cohesion and healthcare utilization, our findings concur with results from a rigorous locally published mixed-methods study carried out by Davidova et al. [62]. In their findings, too, most Roma did not regard low SEP as a stressor; associations between their SEP and self-reported health-related measures were weak; they declared their own health status and the healthcare services they used as acceptable and considered prevention unnecessary; and they viewed familial and local relations with other Roma as their sole resources regarding health.

Our more particular findings regarding specific health-endangering exposures are consistent with and add understanding to findings from related scientific quantitative studies conducted in Slovakia as well as from scientific qualitative studies conducted elsewhere in CEE. This concerns mostly e.g. the previous, then unexpected, findings for Slovakia of equal to lower alcohol-consumption [28, 34], equal to lower promiscuity [35] and equal to higher familial social support and life satisfaction [37, 38] in comparison with local non-Roma. In their qualitative study, conducted with Roma in Bulgaria and Hungary, Kelly et al. [63] found patterns in sexual behaviour similar to those identified by us. Using focus groups to discuss Roma difficulties with access to sexual and reproductive services in Albania, Bulgaria and Macedonia, Colombini et al. [64], too, identified transportation costs and expenses not covered by national health insurance as important barriers.

While compatible with and clarifying some previous findings, our summary also conveys novel information regarding intermediary determinants of health in segregated Roma settlements in Slovakia and beyond. First, the summary provides examples of how particular determinants might vary within the settlements, especially according to social status (see e.g. the differences in perception of low SEP as a stressor). Second, it provides detailed examples of mechanisms by which selected determinants might be contributing to worse health status in the segregated settlements (see e.g. the juxtaposition of particular material circumstances and related health and safety incidents). Third, it demonstrates how specifically particular determinants might be causally inter-linked here (see e.g. the intersectional trade-off relation described between local strategies of coping with stress and local possibilities for acquiring linking and bridging social capital).

Although primarily focused on summarizing the local setup of health-endangering settings and practices, our summary also offers several interesting hints regarding the setup’s possible social root-causes, i.e. regarding why and by whom some of its aspects might be co-maintained. First, it indicates that the found active participation of segregated Roma in the maintenance of particular health exposures might be supported by their broader ethnically understood specific social norms, preferences and tastes (consider e.g. the spontaneous ethnic framing of preferences of relatively well-off Roma for certain exposures). These social norms appeared to be conceived of, developed and maintained by the locals mostly in contrast or direct opposition to respective alleged non-Roma alternatives (consider e.g. the common spontaneous contrasting and down-playing of the ‘non-Roma-like’ ways). Moreover, the appropriateness of the locals’ adherence to the particular norms was often argued by them using racialized arguments (consider e.g. quoting specific related ‘natural’ Roma capacities embodied via their ‘blood’, ‘brains’, ‘bodies’, ‘genes’, etc.).

Second, should the above indication of specific social counter-norms prove correct, it might allow formulations of novel structural constructivist [41] explanations for various previously found surprising associations. E.g. confirmation of distinct understanding on the part of the Roma of their own health-needs, preferences and their own related capacities (see the described informed non-compliance with clinical recommendations within the higher-ranked households despite available required funds) could shed new light on the surprising findings by Geckova et al. [65] and Davidova et al. [62] on the poor correlation between SEP and health-related measures within Roma settlements in Slovakia.

We believe that these two explanatory hints are especially noteworthy, as they suggest the currently discussed range of explanations regarding the persistence of high health exposures segregated CEE Roma face despite a long history of varied interventions [66, 67] might be too narrow. According to their calls for further research, most engaged biomedical researchers seem to expect the health-inequalities between CEE Roma and non-Roma might become fully explained by accounting for ethnic discrimination and social exclusion by non-Roma at the structural level, and for uninformed residual Roma traditions at the level of risky behaviours e.g. [68–70]. Our material strongly indicates that at present in some segregated places a partly racialized ethnically framed active self-exclusion on the part of the Roma might be involved, too.

Historical origin, variability and means of reproduction of such or similar cultural resilience among segregated Roma and analogous groups across the continent is already long being debated in social scientific literature e.g., [16]. Its presence has been realized and continues to be documented especially by ethnographers e.g. [71–75]. But similar findings resonate also in most qualitative research relying on a consistent field-presence and/or more open-ended methods, e.g. in biomedical studies addressing analogous groups beyond CEE e.g. [76, 77] or in other local anthropological research e.g. [78, 79].

### Strengths and limitations

The main strength of our study was its mixed-methods approach. The preliminary socio-graphic survey enabled identification of a relatively typical locality. The use of ethnographic methods enabled intimate access to local everyday settings and local people. The systematic interviewing across several local stratifications allowed accounting for local variability as well as for topical omissions in the previous less-systematic phase. The follow-up communication enabled detection of major changes in the observed phenomena over time and additional reflections of preliminary interpretations by local core informants. Choosing the WHO classification of social determinants of health as a template for analysis and reporting enabled direct public-health significance of our summary according to contemporary health-inequality theories.

Our use of the research-design and our reporting also had some limitations. First, the field-work and most of the analyses were performed by a single researcher, limiting the potential for inter-personal corroboration. Second, although preceded by a careful selection of a place typical for the geographical area, the research examined only a single settlement. Third, it was impossible to remain personally embedded in the settlement for the whole course of the research. Lastly, the explicit focus of our summary on the description of local settings and practices limited the space for presentation of authentic reasoning of the people being described. While all these limitations appear to be well out-weighed by the good match of our findings with other literature, any generalizations of the clues our paper presents should only be attempted with due caution.

## Conclusions and implications

Our summary might serve as detailed and conveniently structured sample material for grounded thinking about health-inequalities within sociologically analogous locations. It offers novel clues especially regarding which intermediary social determinants of health might vary therein and how; which of them might be contributing to health-deterioration and in what way; and how they might be causally inter-linked here.

Our findings also convey that the local preferences for some of the local health-endangering settings and practices were typically framed in racialized ethnic terms constructed in contrast or in direct opposition to alleged non-Roma norms and ways, as such. For public health practice, this indicates that within at least some segregated Roma settlements traditional biomedical interventions and recommendations might be less efficient than elsewhere and why. In cases where the presence of such social counter-norms would apply, the recent trend of designing and implementing public-health interventions using community-based participation may be especially appropriate. To explore this practically and ethically extremely intriguing possibility beyond the limitations of this descriptive summary, more research and analyses are needed focusing directly on the social-root causes of analogous Roma practices.

Our findings also confirm that a social-constructivist approach, i.e. one including a focus on the perspectives of the worse-off populations’ members themselves as well as their broader socio-historical contexts [39, 46, 48], might offer a particularly productive possibility for researching social determinants of health in the case of segregated ethnic communities.

## Appendix

### Basic sociographic parameters^1^

How many people live in the village?

How many people live in the nearby Roma settlement?

How old is the Roma settlement?

Is the settlement physically segregated from the village? If so, how?

What is the situation within the settlement regarding:

- -Water supply
- -Heating
- -Electricity
- -Sewerage
- -Waste disposal
- -Roads

Are there any significant economic and infrastructural differences within the settlement?

How are the social relationships between the villagers and the Roma settlement inhabitants? Are there any restrictions in place in the village regarding the settlement’s inhabitants?

Which general practitioners and pediatricians do the people in the Roma settlement visit?

### Brief questionnaire for local health-care professionals^2^

Are you aware of any activities in place locally focused specifically on the health of the Roma?

Do you consider the health situation of local Roma settlement(s) to be specific?

If so, in what respects?

## Additional file

Additional file 1: Fieldwork visual reference. Illustrative photographs and quotes from the Roma settlement. (PDF 1722 kb)

## Abbreviations

CEE: Central and Eastern Europe
SEP: Socioeconomic position
WHO: World Health Organization
