# Supplementary material for: Health-endangering everyday settings and practices in a rural segregated Roma settlement in Slovakia: A descriptive summary from an exploratory longitudinal case study
Source: BMC Public Health. 2017 Jan 28;17:128. doi: 10.1186/s12889-017-4029-x (PMC5273804; doi:10.1186/s12889-017-4029-x)
Supplement: Additional file 1: — Fieldwork visual reference. Illustrative photographs and quotes from the Roma settlement. (PDF 1722 kb) [file 12889_2017_4029_MOESM1_ESM.pdf]

Material circumstances

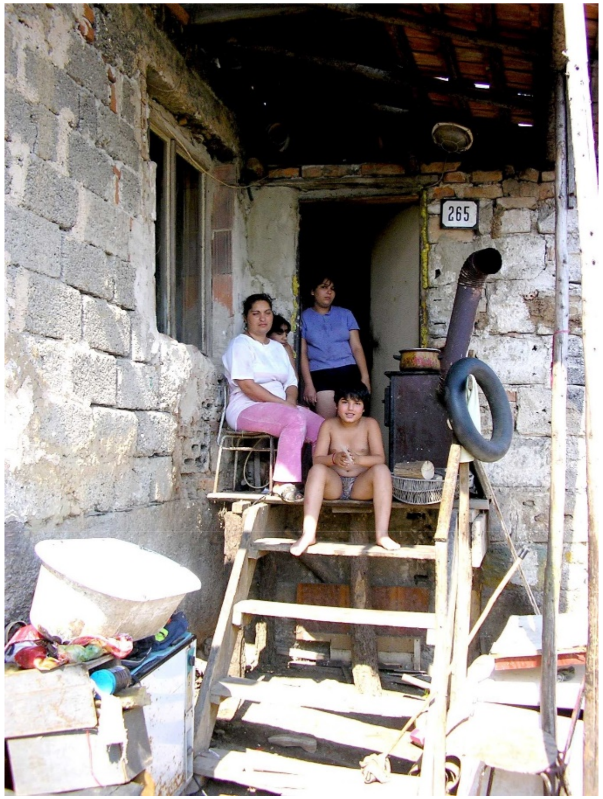

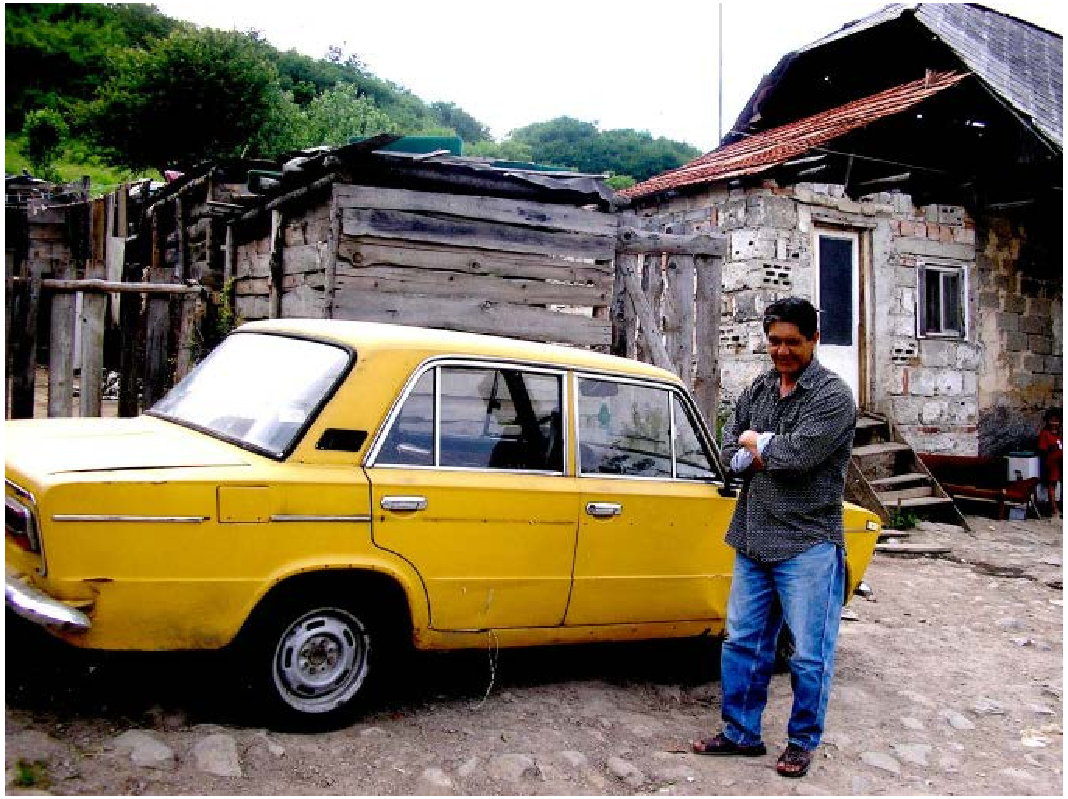

‘*I wish they* [own children] *would have more money than us… Why? So they’re not down like us, so they don’t have to steal wood, recycle metal.*’ A., woman, 34, low social level [Sep 2005]

‘*Do you know why Roma have always preferred the Žigulis over the Škodas* [car brands]? *Because of their acceleration! A Gypsy needs his engine to roar, you know what I mean?*’ Z., man, 37, medium social level [Jul 2005]

Psychosocial factors

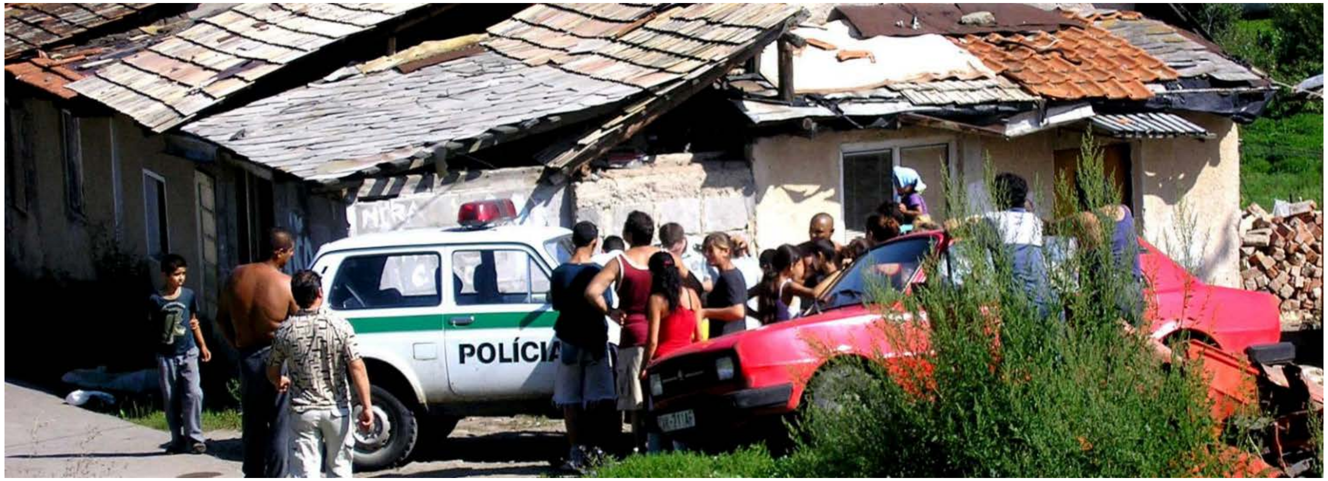

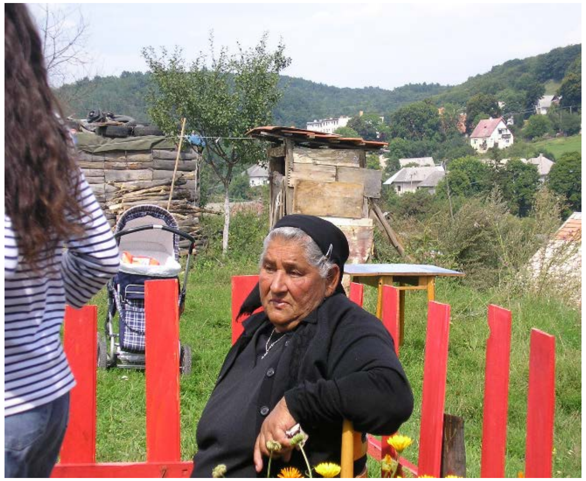

‘*I tell you why* [many Roma nowadays visit psychiatrists for subscription medications]. *Because these are the hardest times we’ve ever been through. It has never been this bad before. Everybody can feel nothing good is coming our way anymore. Women are afraid for their kids’ future.* [Seasonal] *work for men is gone. We will now even be working for free* [referring to a new unemployment law]!’ S., man, 41, medium social level [Aug 2014]

This is the hardest thing both for him [referring to husband and father of four away at a rehab stay] *and us* [his nuclear family and siblings]. *We don’t see him every day, we can only visit him now and then thanks to M* [a better off sister]. *He’s among the non-Roma all by himself all the time. The terrible silence, the watery food. Oh, God, I really think it would be far better for all of us if he just stayed at home and drank himself to death here!’* K., woman 43, medium social level [Jul 2005]

Health-related behaviour

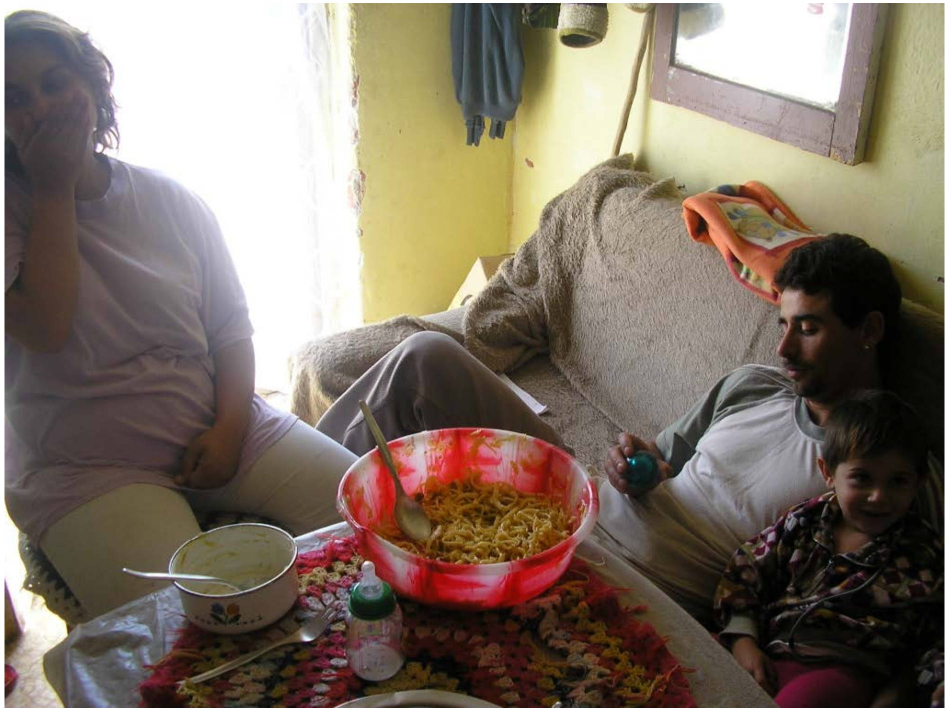

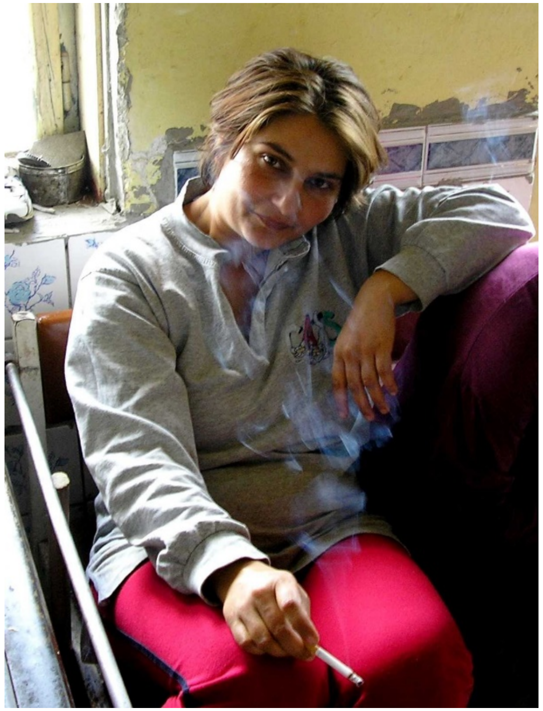

‘*Gypsies love meat, especially pork meat, everybody knows that.* […] *And we hate thin food with no taste. The soups you eat, pure water!*’ M2, woman, 27, high social level [Sep 2008]

‘*Of course I believe it's true* [that smoking causes cancer]. *So what? Nobody will ever make me stop anyway. A Gypsy* [sic] *will always only want to live like a Gypsy... this is his life, to have a little smoke, to have a little fun, to drink a little.*’ S., man, 31, medium social level [Jun 2005]

Social cohesion

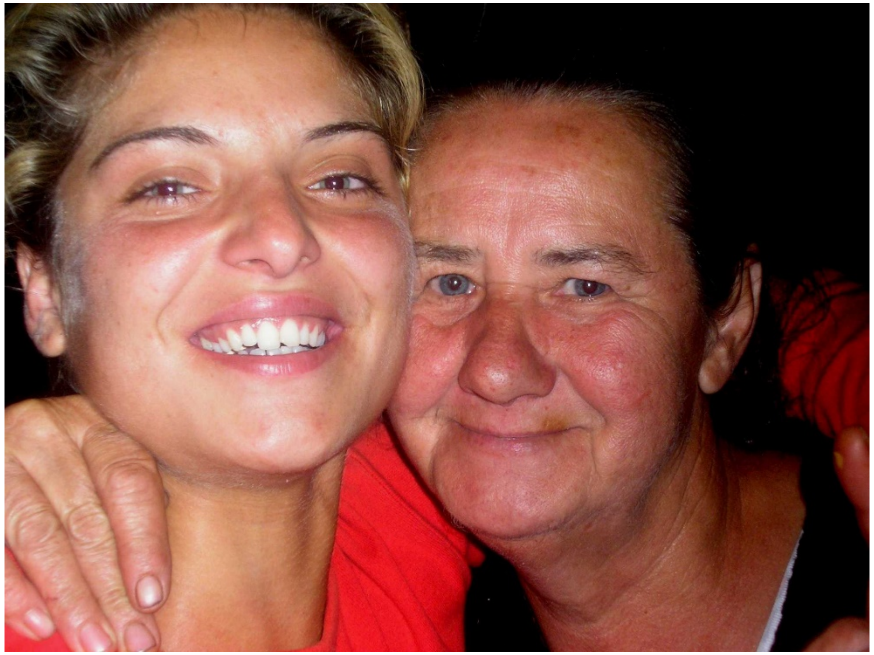

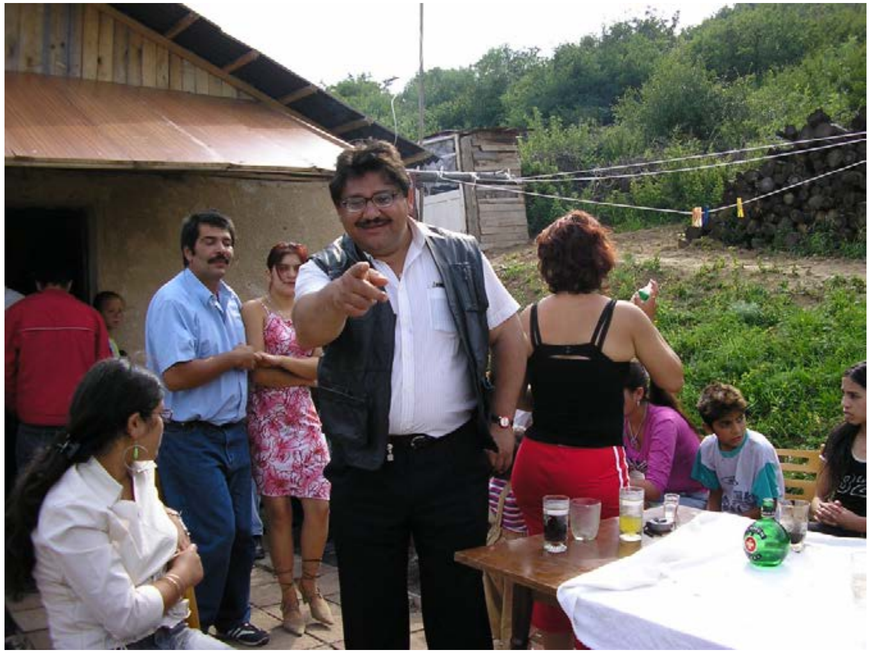

‘[If their kids would grow-up at A.B.’s mother’s house], *it would still be the same for them, I think. Maybe some small things would change… for some time… but most of it would be the same. It’s in our blood! You know how they say ’Gypsy blood’ – you cannot change that, no matter what.* […] *Our life is so much better, more colourful.*’ L., woman, 43, high social level [Sep 2014]

‘*I don't understand how you non-Roma can let your little children be treated in schools the way they are being treated there. Strangers yelling at them... all the boring stuff... and you have to sit there and sit silently forever. Roma kids are unable to go through that... your kids are different.*’ M., woman, 36, high social level [Mar 2010]

Healthcare use

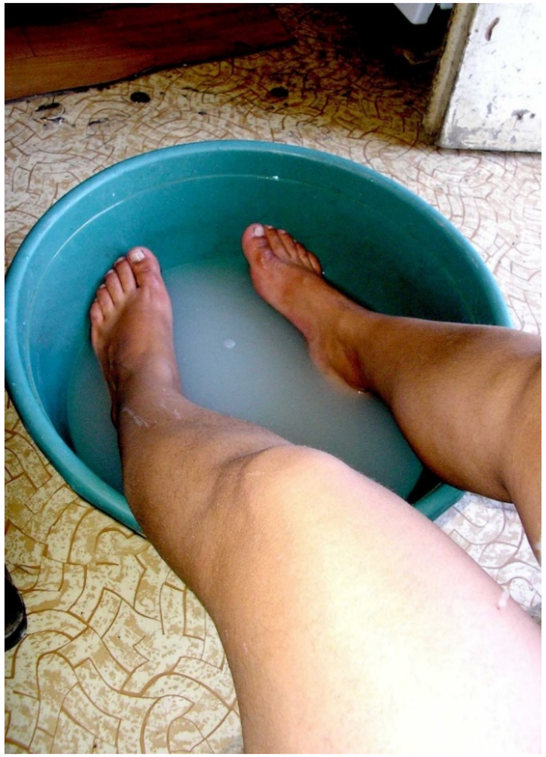

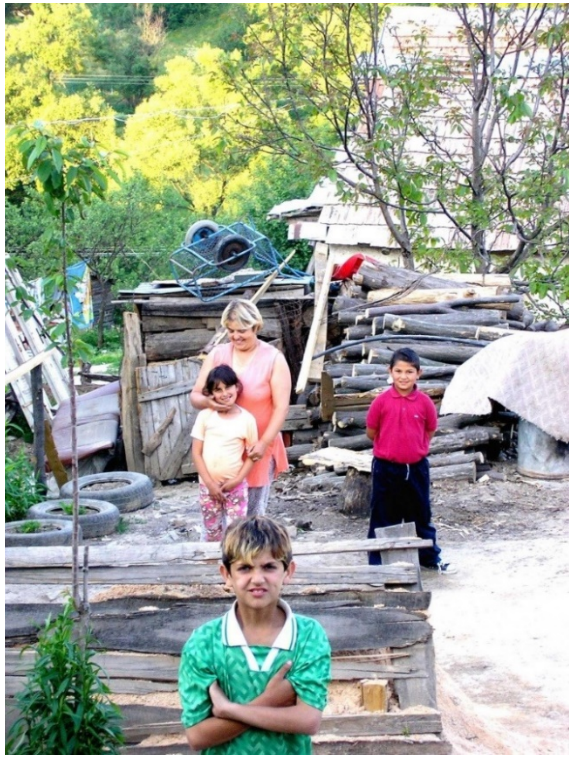

‘*You bet I’d see the doctor more often* [did she have more cash for public transport]… *with my kids at least* […] *How would we take those* [prescription pills]*? We take them exactly as told* [by the healthcare staff]… *sure, the way the hours are supposed to go* […] *Are you crazy? You cannot just add more to what you should be taking, that could hurt you… or it just doesn’t work then.*’ A., woman, 34, low social level [Sep 2005]

‘*Of course they* [healthcare staff in the nearby administrative centre] *treat non-Roma differently… but they don’t do any harm to us.*’ K., man, 27, medium social level [Sep 2005]
